# CBX7 is a glioma prognostic marker and induces G1/S arrest via the silencing of CCNE1

**DOI:** 10.18632/oncotarget.15789

**Published:** 2017-03-01

**Authors:** Tianfu Yu, Youzhi Wu, Qi Hu, Junxia Zhang, Er Nie, Weining Wu, Xiefeng Wang, Yingyi Wang, Ning Liu

**Affiliations:** ^1^ Department of Neurosurgery, The First Affiliated Hospital of Nanjing Medical University, Nanjing 210029, China; ^2^ Department of Neurosurgery, Nanjing First Hospital, Nanjing Medical University, Nanjing 210006, China; ^3^ Department of Neurosurgery, First People's Hospital of Yueyang, Yueyang 414000, China

**Keywords:** CBX7, epigenetics, cell cycle, glioma

## Abstract

Chromobox homolog 7 (CBX7) cooperates with other polycomb group (PcG) proteins to maintain target genes in a silenced state. However, the precise role of CBX7 in tumor progression is still controversial. We found that the expression of CBX7 in four public databases was significantly lower in high grade glioma (HGG). The reduced expression of CBX7 correlated with poor outcome in HGG patients. Both KEGG and GO analyses indicated that genes that were negatively correlated to CBX7 were strongly associated with the cell cycle pathway. We observed that decreased CBX7 protein levels enhanced glioma cells proliferation, migration and invasion. Then, we verified that CBX7 overexpression arrested cells in the G_0_/G_1_ phase. Moreover, we demonstrated that the underlying mechanism involved in CBX7 induced repression of *CCNE1* promoter requiring the recruitment of histone deacetylase 2 (HADC2). Finally, *in vivo* bioluminescence imaging and survival times of nude mice revealed that CBX7 behaved as a tumor suppressor in gliomas. In summary, our results validate the assumption that CBX7 is a tumor suppressor of gliomas. Moreover, CBX7 is a potential and novel prognostic biomarker in glioma patients. We also clarified that CBX7 silences *CCNE1* via the combination of *CCNE1* promoter and the recruitment of HDAC2.

## INTRODUCTION

Chromobox homolog 7 (CBX7) is a chromobox family protein which belongs to polycomb repressive complex 1 (PRC1). In cooperation with other polycomb group (PcG) proteins, CBX7 maintain target genes, which participate in stem cell self-renewal and developmental regulation, in a silenced state [1–4]. CBX7 recognizes H3K27me3, which is catalyzed by enhancer of zeste homolog 2 (EZH2). Then, CBX7 recruits other PRC1 proteins to the target chromatin marked by H3K27me3 and represses gene transcription consequently [5–7]. Besides the PRC-dependent mechanism, recent studies found that CBX7 epigenetically regulates gene expression via the interaction with histone deacetylase 2 (HADC2) [8, 9]. However, the precise role of CBX7 in tumorigenesis and tumor progression is still controversial.

Gil and colleagues first identified CBX7 and found that the lifespan of differentiated human cells was extended and the immortalization of mouse fibroblasts was established via increased CBX7 levels which was followed by downregulating expression of the Ink4a/Arf locus [1, 2]. CBX7 also cooperated with c-Myc and initiated lymphomagenesis [4]. However, of note, CBX7 expression has a negative correlation with advanced tumor aggressiveness and poor prognosis in a wide range of tumor types, such as thyroid, colorectal, pancreas, breast, lung, and glioblastoma (GBM) [9–18]. The contradictory behavior of CBX7 in different kinds of tumors reflects the notion that cellular context plays an essential role in the effects of the CBX7.

GBM, a grade IV astrocytoma that originates in the brain, is the most aggressive and common primary neoplasm [19]. Owing to short median survival time (around 12–15 months) and poor prognosis, elucidation of molecular mechanisms in GBM is urgently needed [20]. Although it was reported that CBX7 was downregulated and inhibited cell migration in glioma cells, the effect of CBX7 on cell cycle disregulation, another typical characteristic of GBM, is still unclear. In this study, we tested the relationship among CBX7 mRNA level, tumor grade and poor prognosis in CGGA, TCGA, REMBRANDT and GSE16011 datasets. We found that CBX7 possessed prognostic value in glioma patients. Then bioinformatics analysis indicated that CBX7 played a tumor suppressor role in glioma progression, especially in cell cycle control. CBX7 overexpression impaired the proliferation, migration and invasion of GBM cells; downregulation of CBX7 had the opposite effects. Furthermore, CBX7 inhibited G_1_/S phase transition via binding and silencing *CCNE1* promoter mediated by the recruitment of HDAC2. Finally, CBX7 attenuated tumor growth in a xenografted model. Taken together, CBX7 participates in G_1_/S checkpoint control and is a novel prognostic marker in glioma patients.

## RESULTS

### Decreased CBX7 expression correlates with glioma grade and its overexpression confers a better prognosis in HGG patients

**Figure 1:**
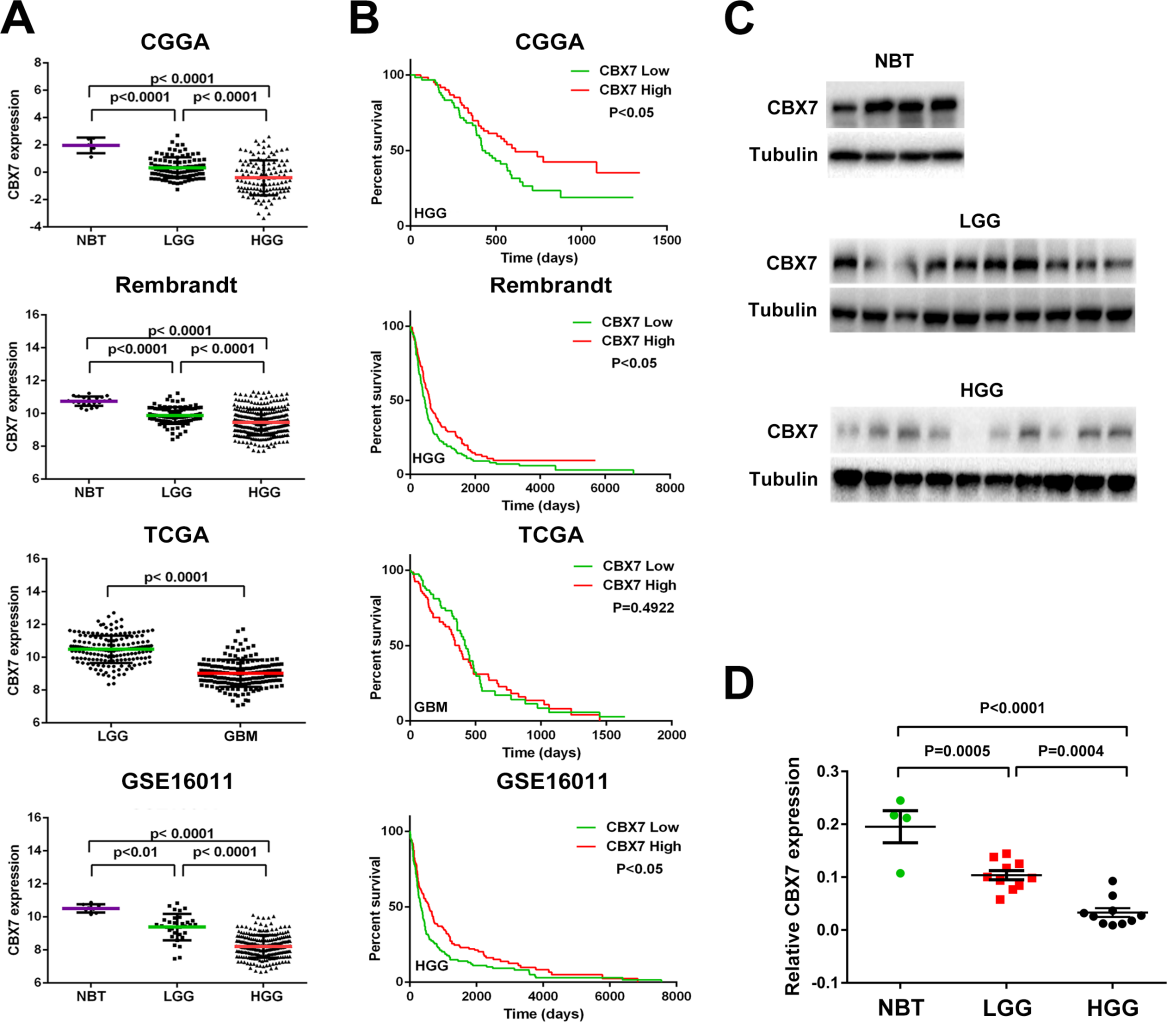
CBX7 mRNA level is decreased accompanied by the elevation of tumor grade and low expression of CBX7 is associated with poor prognosis in part of glioma cases (**A**) The transcriptional levels of CBX7 were tested in CGGA, TCGA, REMBRANDT, and GSE16011 glioma datasets. (**B**) Kaplan-Meier survival curve was used to estimate the survival outcomes of patients divided into two groups with CBX7 low/high level. (**C**) Western blot analysis was used to test the levels of CBX7 in different glioma samples and normal brain tissues. Tubulin was used as a loading control. (**D**) Q-PCR was performed to determined CBX7 levels among NBT, LGG and HGG samples.

Considering the contradictory role of CBX7 in different tumors, the transcription level of CBX7 in four glioma databases (CGGA, TCGA, REMBRANDT and GSE16011) was analyzed. We found that CBX7 mRNA levels in high-grade gliomas (HGG) were significantly decreased compared to normal brain tissues (NBT) and low-grade gliomas (LGG). CBX7 expression was also decreased in LGG in comparison to NBT (Figure 1A). Further, the association between CBX7 mRNA levels and clinical progression of glioma patients was determined using overall survival rates in GBM patients (samples from TCGA) and HGG patients (samples from CGGA, REMBRANDT and GSE16011), and Kaplan-Maier analysis and log-rank comparison were performed. In TCGA database, there was no significant difference in clinical outcome between the high and low CBX7-expression groups (high versus low median survival, 418 versus 375 d, respectively; *p* = 0.4922). However, in the three other databases, decreased CBX7 expression was associated with a shorter survival period (Figure 1B). Twenty human glioma specimens were divided into LGG or HGG groups and four brain samples obtained from epilepsy surgery were used for control group. In these samples we found that protein and mRNA levels of CBX7 are also negatively associated with tumor grade (Figure 1C and 1D). We also found that CBX7 expression possesses subtype preferences within gliomas (Supplementary Figure 1). These multi-center data and tumor samples revealed that CBX7 is a potential prognostic factor in glioma patients.

### CBX7 associated genes are mainly enriched in cell division cycle pathways

**Figure 2:**
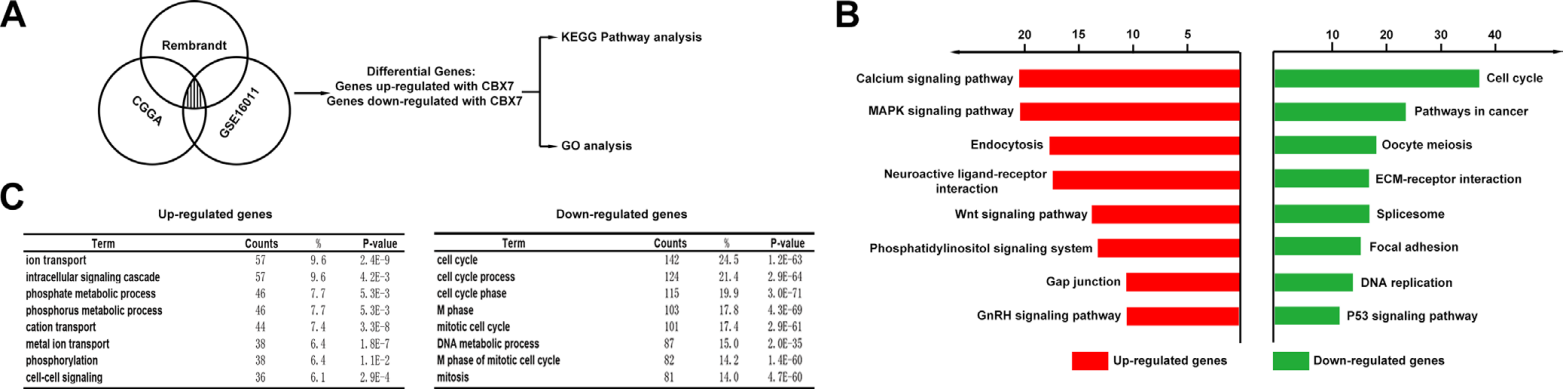
The CBX7-associated genes were chiefly enriched in cell cycle related pathways (**A**) CBX7 associated genes obtained from overlapping CGGA, GSE16011 and Rembrandt databases via correlation analysis were analyzed with KEEG pathway analysis and GO enrichment analysis. (**B**) Enrichment results of KEGG pathways analysis were shown in different colours (red stands for up-regulated genes and green represents down-regulated genes). (**C**) Biological processes enrichment results from GO database. The orders of different biological processes were based on their enriched number.

We performed a Pearson correlation analysis to discover genes that were associated with CBX7 in CGGA, Rembrandt and GSE16011 datasets (R > 0.4). To clarify the associations between these genes and specific GO functional categories, DAVID Web tool (http://david.abcc.ncifcrf.gov/home.jsp) and KEGG pathway analysis were used. In total 594 upregulated genes and 579 downregulated genes were identified (Figure 2A). As is shown in Figure 2B, decreased gene expression profiles were more enriched in pathways related to cell cycle and cancer signaling pathways. Furthermore, GO enrichment analysis also showed that these downregulated genes were strongly enriched in the cell cycle regulation process (Figure 2C). The in silico prediction of CBX7 function was in line with tumor-associated phenotypes.

### Regulation of CBX7 expression influences the malignant behavior of GBM cells

**Figure 3:**
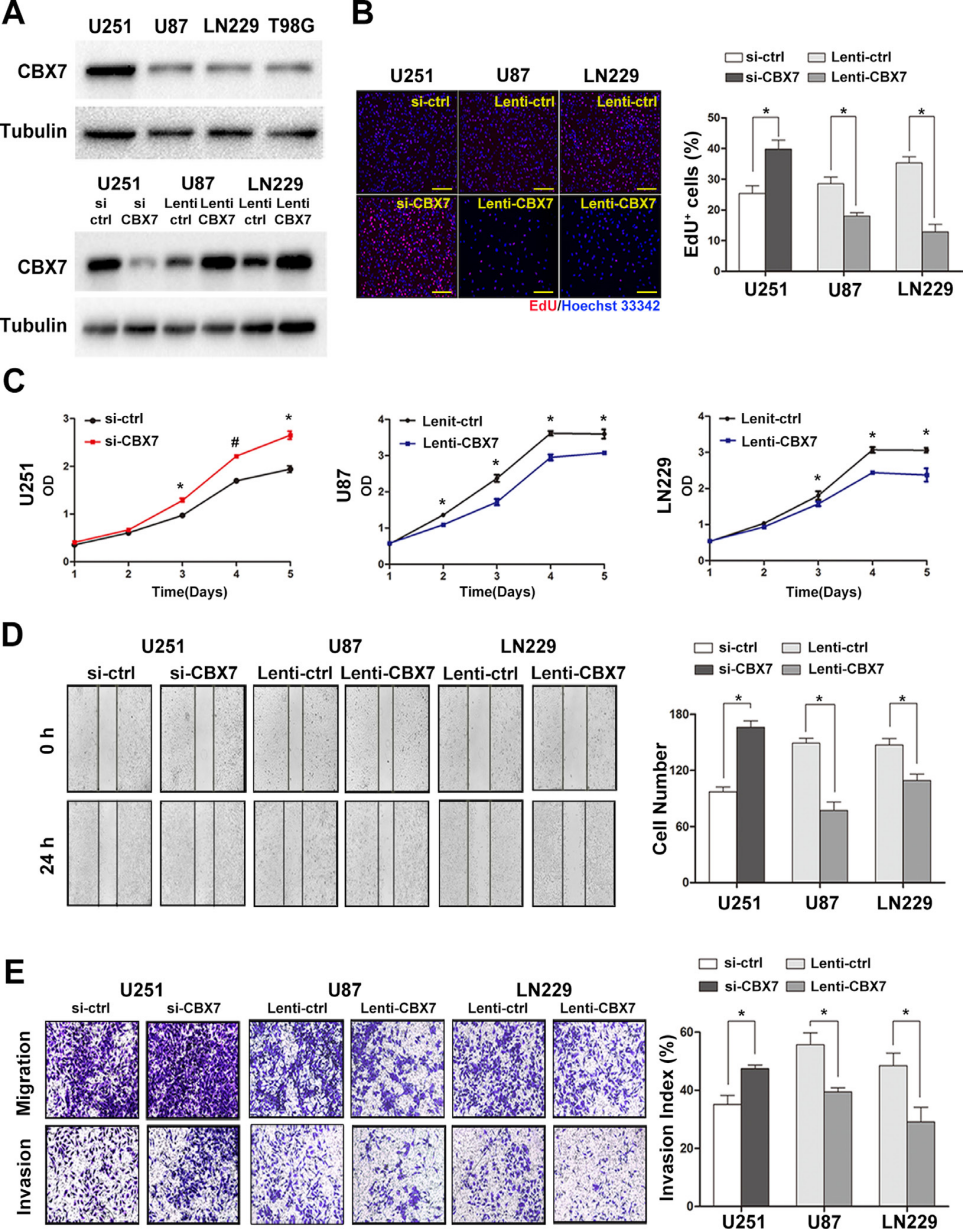
The up or downregulation of CBX7 expression by lentivirus or siRNAs impacts the proliferation, migration and invasion of GBM cells *in vitro* (**A**) The protein levels of CBX7 in four classic GBM cell lines were examined through western blot assay (up panel). After transfection with siRNAs or lentivirus vectors, CBX7 expression in three GBM cell lines was assessed by western blot (down panel). (**B**) Respective merged images of U251, U87 and LN229 cells transfected with siRNAs or lentivirus vectors. Data are means of three independent experiments + SEM. *P < 0.05. Scale bar = 200 mm. (**C**) Proliferative abilities of GBM cells infected with siRNAs or lentivirus vectors were tested via CCK8 assays. *P < 0.05, ^#^*P* < 0.001. (**D**, **E**) Wound-healing assay and transwell assay were performed to evaluate the migrative and invasive ability of GBM cells transfected with siRNAs or lentivirus vectors. *P < 0.05, ^#^*P* < 0.001.

Bioinformatics database-based results demonstrated that CBX7 was closely related to the progression and prognosis of gliomas and participated in cell cycle regulation. However, this in silico evidence was insufficient to elucidate the role of CBX7 reliably. Therefore, we constructed siRNAs targeting CBX7 and a lentivirus vector expressing CBX7. We then assessed the CBX7 protein levels in four GBM cell lines (Figure 3A). Other typical PRC1 elements in U251, U87 and LN229 cells were also tested (Supplementary Figure 2A). U251 cells containing high concentrations of CBX7 were used in knockdown experiments of CBX7 expression via the transient transfection of siRNA targeting CBX7 (si-CBX7) as well as a control vector (si-ctrl). The efficiency of three different sequences si-CBX7 was tested in U251 cells and the optimal si-CBX7 sequences was chosen for the subsequent experiments (Supplementary Figure 2B). We further constructed two stable CBX7 expressing cell lines, U87 and LN229, using a lentiviral CBX7-expression vector (Lenti-CBX7) and control cell lines using a control lentiviral vector (Lenti-ctrl) in the parental cells. The efficiency of up/downregulation of CBX7 is shown in Figure 3A.

To explore the biological function of CBX7 in glioma cells, EdU, CCK8, wound-healing and transwell assays were performed to study the influence of CBX7 on cell proliferation, migration and invasion. After incubation with si-CBX7 for 48 h, the growth rate of U251 cells was increased. Conversely, the two stable CBX7 expressing cell lines, U87 and LN229, showed impairment in proliferation capability (Figure 3B and 3C). A known malignant behavior of glioma cells is its strong tendency for local invasion. We found that si-CBX7 treatment potentiated tumor aggressiveness in U251 cells compared with si-ctrl treatment. Conversely, the migrative and invasive capability in CBX7-overexpressing cell lines was suppressed (Figure 3D and 3E). In order to eliminate the influence of differences among cell types, we performed part of experiments in U251 with the use of siRNA and lentiviral vector and the results were consistent with the previous studies (Supplementary Figure 2C–2F). These findings imply that CBX7 is a potential tumor suppressor gene in glioma cells.

### CBX7 binds and silences *CCNE1* promoter mediated by the recruitment of HDAC2 and leads to G_1_/S arrest

**Figure 4:**
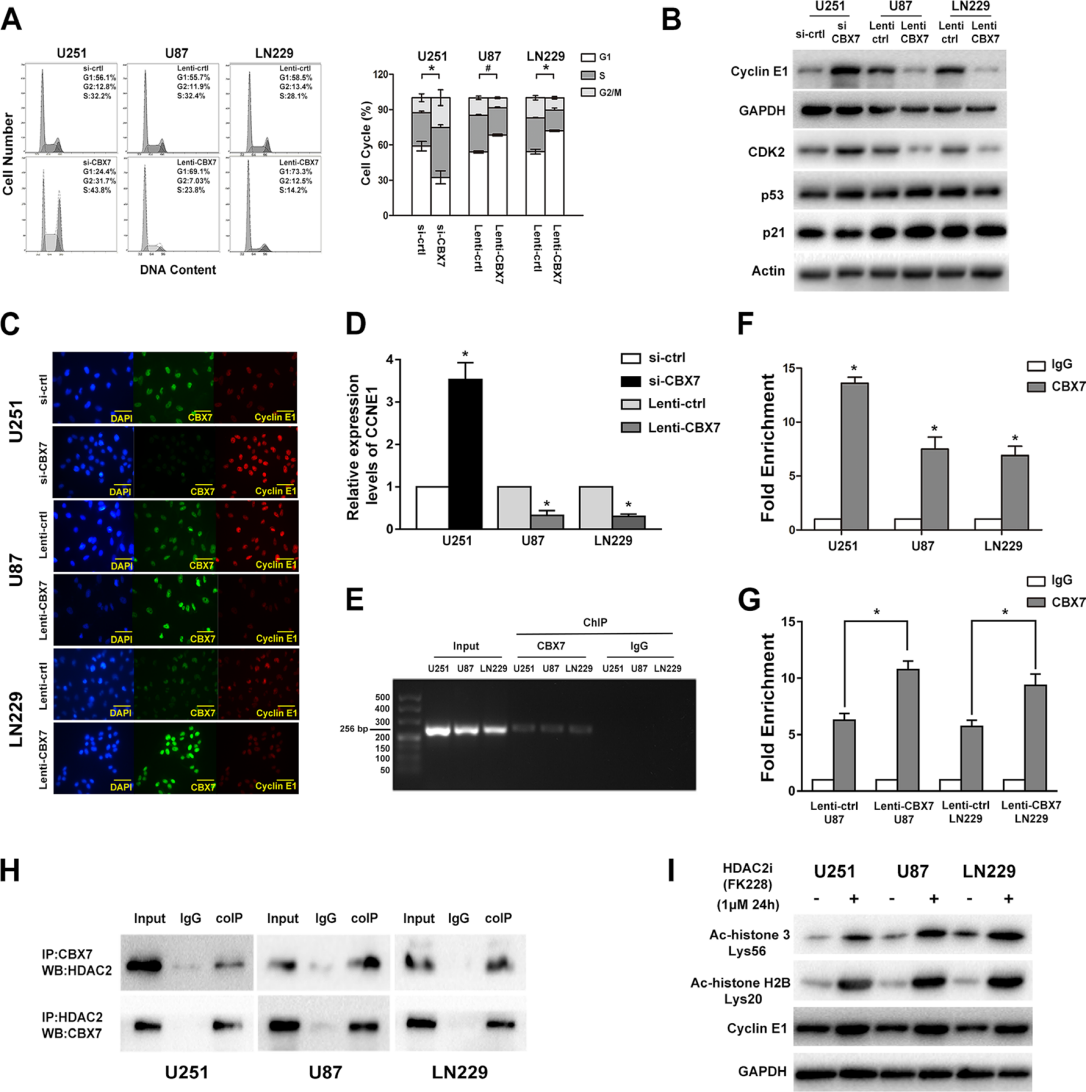
CBX7 induces G_1_/S arrest through binding and silencing CCNE1 promoter which mediated by the recruit of HDAC2 (**A**) Flow cytometic assay was used to assess the cell cycle in GBM cells transfected with siRNAs or lentivirus vectors and the right panel showed the percent of different cell cycle phases. (**B**) Cyclin E1, CDK2, p53 and p21 levels were tested after the regulation of CBX7 in different GBM cell lines. (**C**) Immunofluorescence staining of CBX7 (green) and Cyclin E1 (red) in in different GBM cells. Scale bar = 50 mm. (**D**) Relative expression of *CCNE1* in CBX7 up/down regulated cells was studied by Q-PCR. *P < 0.05. (**E**, **F**) ChIP experiment was used to examine the interaction between CBX7 and *CCNE1* promoter. The precipitate was collected and evaluated by both RT-PCR and q-PCR. *P < 0.05, ^#^*P* < 0.001. (**G**) ChIP assay showed that the occupancy of *CCNE1* promoter by CBX7 was increased in CBX7 overexpression U87 and LN229 cells. *P* < 0.05. (**H**) Co-IP analysis was performed to evaluate the interaction of CBX7 and HDAC2. Total cell lysates from GBM cells were co-immunoprecipitated by anti-CBX7 antibody and anti-HDAC2 antibody. Protein-antibody complexes were analyzed by western blot and the results showed that CBX7 binds to HDAC2 in three cell lines. (**I**) After treated with 1μM HDCA2 inhibitor FK228 for 24 h, the degree of histone acetylation (acetyl-histone H3 Lys56 and acetyl-histone H2B Lys20) and cyclin E1 levels in three GBM cell lines were tested via western blot.

Our bioinformatics analysis indicated that CBX7-associated genes were enriched in cell cycle-related regulators. Moreover, Fusco and colleagues reported that CBX7 bound to the *CCNE1* promoter as a multiprotein complex that included HDAC2 and then suppressed *CCNE1* expression in mice and HEK293 cells [9]. Thus, we emphasized analyzing the influence of CBX7 on the cell cycle and the mechanism by which CBX7 regulates *CCNE1* expression. Flow cytometry analysis showed that the downregulated CBX7 levels promoted G_1_/S transition, which was halted by the overexpression of CBX7 (Figure 4A). The levels of cyclin E1, CDK2, p53 and p21 were meansured using western blotting (Figure 4B). Depleted CBX7 levels triggered an improvement in cyclin E1 and CDK2 levels, while the restoration of CBX7 expression lead to decreased cyclin E1 and CDK2 expression (Figure 4C). We also found that the expression of *CCNE1* was regulated by CBX7 at the transcriptional level (Figure 4D).

Chromatin Immunoprecipitation (ChIP) assays were conducted to test whether the CBX7 could bind to the *CCNE1* promoter in glioma cells. Protein-chromatin complexes were subjected to immunoprecipitation with anti-CBX7 or anti-IgG antibodies and then the precipitate was analyzed using reverse transcription-PCR (RT-PCR) and quantitative real-time-PCR (q-PCR) (Figure 4E and 4F). Then, U87 and LN229 cells with exogenous expression CBX7 protein were used to further assess the interaction between CBX7 and *CCNE1* promotor. Along with the increased CBX7 levels, the enrichment of precipitated *CCNE1* promotor fragment was ascending (Figure 4G). To verify the interaction between CBX7 and HDAC2, lysates of U251, U87 and LN229 cells were subjected to IP with anti-CBX7 antibodies or nonspecific IgG. The immunocomplexes were immunoblotted with anti-HDAC2 antibodies. Conversely, anti-HDAC2 antibodies were used to pull down the proteins combine to HDAC2. Then, the immunocomplexes were immunoblotted with anti-CBX7 antibodies to ascertain that HDAC2 combines to CBX7. Using co-immunoprecipitation (coIP) assays, we confirmed that CBX7 interacted with HDAC2 (Figure 4H). We tested the degree of histone acetylation and cyclin E1 levels in three GBM cell lines treated with 1μM HDCA2 inhibitor FK228 for 24 h and found that inhibition of HDAC2 rescued the levels of cyclin E1 (Figure 4I).

These data verified that CBX7 induces G_1_/S arrest via the repression of *CCNE1* promoter transcriptional activity and that this mechanism is mediated by HDAC2.

### Overexpression of CBX7 in U87 cells impairs orthotopic tumor growth *in vivo*

**Figure 5:**
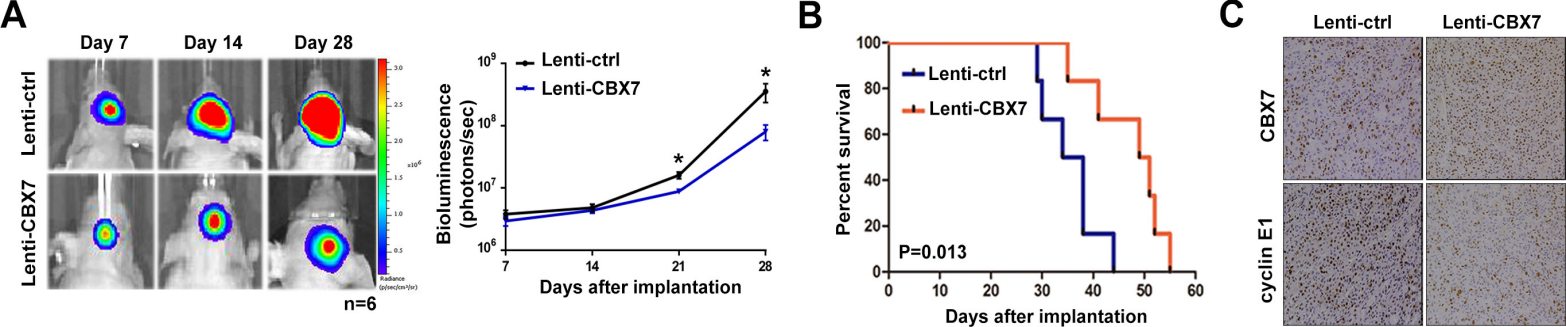
The upregulation of CBX7 protein level leads the growth suppression of intracranially xenografted GBM and prolongs the survival time of nude mice (**A**) Tumor volumes (*n* = 6 mice/group) were assessed by noninvasive bioluminescence imaging system and representative images taken at different days were shown in left panel. Quantification of the bioluminescence signal was plotted in right panel. *P* < 0.05. (**B**) Overall survival was calculated through Kaplan-Meier survival curves and the log-rank test was applied to estimate *p*-value between two groups. (**C**) IHC sections came from tumor slices were stained with CBX7 and Cyclin E1 and the representative profiles were demonstrated.

An orthotopic mouse model using U87 cells was performed to further determine the role of CBX7 in gliomagenesis. U87 cells were initially transfected with luciferase expressing lentiviruses and then these cells were transfected with Lenti-ctrl or Lenti-CBX7. After implantation of U87 cells in mice, intracranial tumor volume was measured weekly via *in vivo* optical imaging system. Results from bioluminescence imaging showed that growth of glioma was significantly suppressed by CBX7 overexpression (Figure 5A). Concomitant with inhibition of tumor formation, mice treated with Lenti-CBX7 had prolonged survival times (Figure 5B). In addition, immunohistochemistry (IHC) revealed that upregulated CBX7 was accompanied by decreased cyclin E1, which was consistent with the *in vitro* results (Figure 5C). In summary, these findings showed that CBX7 inhibits glioma cell proliferation *in vivo*.

## DISCUSSION

Epigenetic alterations such as histone modifications and DNA methylation broadly participate in biological functions and gene regulation, and are essential mechanisms in cancer development apart from genetic abnormalities [21, 22]. PcG proteins act as epigenetic regulators of gene expression and play pivotal roles in both physiological and pathological cellular activities [23–32]. CBX7 participates in the interaction between PRC2 and PRC1; another two PcG elements, EZH2 and BMI-1 belonging to PRC2 and PRC1, respectively, are described as oncoproteins [33–35]. However, the role of CBX7 in malignancy is still inconclusive. Our bioinformatics analysis has revealed that CBX7 is strongly linked to control of cell division. Indeed, CBX7 has been shown to repress Ink4a/Arf locus and impair p14^Arf^/p53- and p16^Ink4a^/Rb-dependent pathways in prostate cancer, human follicular lymphomas and gastric cancer [1, 2, 4, 36]. However, Ink4a/Arf locuses in U251, U87, and LN229 cells undergo homozygous deletion and the expression of p16^Ink4a^ in HGG is abrogated [37]. This phenomenon partially impair the oncogenic effect of CBX7. Thus CBX7 may possesse dual functions in cell cycle regulation and maintain a balance between cell division and quiescence. We tested p16 levels in three types of patient-derived primary GBM cells (G1, G2 and G3) and one p16-positive cell type (G1) was found (Supplementary Figure 3A). Compared to NHA, the CBX7 level in G1 was similar but G1 had a higher level of cyclin E1 and a lower level of p16 (Supplementary Figure 3A). After the upregulation of CBX7, the p16 expression was decreased and the cyclin E1 level was impaired (Supplementary Figure 3B). Interestingly, cyclin E1 abundance showed no significant difference in siRNA or lentivirus vector transfected NHA cells. By contrast, p16 protein levels were obvious changed along with the alteration of CBX7 in NHA (Supplementary Figure 3B). We suspect that the expression of CCNE1 in normal cells such as NHAs has been fully inhibited so that the regulation CBX7 cannot further effect CCNE1 expression. In cancer cells, cyclin E1 levels are up-regulated and, conversely, p16 levels was limited via various ways so that CBX7 acts as a tumor repressor in part of GBM cells. We assumed that this balance favors cell quiescence, owing to the anomalous genetic characteristics in GBM. To address this issue, we tested the function of CBX7 in glioma *in vitro* and *in vivo*.

In our study, we showed that CBX7 acted as a glioma suppressor via G_1_/S arrest. First, we analyzed CBX7 mRNA levels and patient outcomes in four public databases including CGGA, TCGA, REMBRANDT and GSE16011 databases. In all cohorts, the expression of CBX7 was significantly lower in HGG compared with LGG or NBT. The result showed that there was a significant inverse correlation between CBX7 level and glioma grade. In addition, survival analysis portrayed CBX7 as a valuable biomarker in survival prediction because the reduced expression of CBX7 correlated with poor outcome in HGG patients.

Three datasets were overlapped to mine CBX7-associated biological pathways. Both KEGG and GO analyses indicated that genes that were negatively correlated to CBX7 were strongly associated with the cell cycle pathway. Next, three GBM cell lines with different CBX7 abundance were chosen to further study the biological functions of CBX7. We observed that increased CBX7 protein levels inhibited glioma cells proliferation, migration and invasion. Then, we verified that CBX7 overexpression arrested cells in the G_0_/G_1_ phase. Moreover, we demonstrated that the underlying mechanism in CBX7 repression of *CCNE1* promotor activity requiring HDAC2 recruitment and resulting in impaired *CCNE1* transcription. Finally, orthotopic glioma models in nude mice implanted with CBX7-overexpressing U87 cells were used. Non-invasive bioluminescence imaging and survival times of nude mice revealed that CBX7 behaved as a tumor suppressor gene in gliomas.

The interaction between cyclin E and CDK2 plays an essential role in the G_1_/S phase transition via phosphorylation of p27^Kip^. Misregulation of cyclin E/CDK2 complex occurs in various types of tumors and leads to uncontrolled cell growth [38–40]. Thus, the CBX7-induced suppression of *CCNE1*, which encodes cyclin E1, contributed to the attenuation of glioma progression. Considering the complexity of phosphorylated proteins downstream of the cyclin E/CDK2 complex, further investigation is warranted. Furthermore, the interaction between CBX7 and other PcG proteins such as EZH2, CBX6 and CBX8 will require additional clarification.

In conclusion, our *in vitro* and *in vivo* results validate the assumption that CBX7 is a tumor suppressor of gliomas. Moreover, CBX7 is a potential and novel prognostic biomarker in glioma patients. In addition to showing CBX7-induced phenotypic changes, we also found that CBX7 silences *CCNE1* via the combination of *CCNE1* promoter and the recruitment of HDAC2.

## MATERIALS AND METHODS

### Public datasets

Whole genome mRNA expression microarray data and clinical information of 220 glioma samples and 5 normal brain tissues were obtained from Chinese Glioma Genome Atlas (CGGA) database (http://www.cgga.org.cn) and used as discovery set [19]. Three validation sets are The Cancer Genome Atlas database (TCGA, http://cancergenome.nih.gov), Repository of Molecular Brain Neoplasia Data (REMBRANDT, https://caintegrator.nci.nih.gov/rembrandt/) and GSE16011 data (http://www.ncbi.nlm.nih.gov/geo/query/acc.cgi?acc=GSE16011).

### Cell culture and treatment

Human glioblastoma cells (LN229, U87, and U251) were purchased from the cell bank of the Chinese Academy of Sciences (Shanghai, China). Primary GBM cells (G1, G2 and G3) that derived from three primary GBM surgical specimens were maintained in DMEM supplemented with 10% FBS. Normal human astrocytes were purchased from ScienCell Research Laboratories (USA). Three different sequences of CBX7 siRNA were purchased from GenePharma (Shanghai, China). Lentivirus vectors and plasmids were purchased from GeneChem (Shanghai, China). FK228 was purchased from MedChemExpress (USA).

### Patient tumors

Four normal brain tissues, ten primary LGG and ten HGG samples were obtained from Department of Neurosurgery, the First Affiliated Hospital of Nanjing Medical University (Nanjing, China). The clinic pathological and general information of those patients are shown in Supplementary Table 1. Written informed consent was obtained from all patients. Our study was approved by the Institutional Review Board and the ethics committee of Nanjing Medical University and Harbin Medical University.

### Western blot analysis

RIPA lysis buffer (KeyGEN, Jiangsu, China) was used to extract proteins from cells. Equal amounts of protein were separated by SDS-PAGE, followed by electrotransfer onto polyvinylidene difluoride membranes (Thermo Fisher Scientific, Massachusetts, USA). Immunoblot analysis used the following primary antibodies: CBX7 (ab21873), HDAC2 (ab12169), Cyclin E1 (ab3927), p16 (ab51243), p53 (ab28), Actin (ab8226), Tubulin (ab7291) and GAPDH (ab9485) were obtained from Abcam (Cambridge, UK). P21 (#2946), acetyl-histone H3 (Lys56) (#4243), acetyl-histone H2B (Lys20) (#2571) were purchased from Cell Signaling Technology (Massachusetts, USA).

### EdU proliferation assay

Click-iT EdU Alexa Fluor 594 Imaging Kit (Thermo Fisher Scientific, Massachusetts, USA; Cat. No. C10339) was used according to the manufacturer's protocol.

### CCK-8 assay

After treatment with siRNA or lentivirus, cell growth was measured by CCK-8 Cell Counting Kit (Dojindo, Japan) following the manufacturer's protocols.

### Wound-healing assay

SiRNA or lentivirus transfected cells were seeded in 6-well plates and cultured until reached 100% confluence. Then, sterile pipette tips were used to make scratches across the cell monolayer and the photographs of scratched areas were taken. 24 h later, different scratched areas were selected in each well and were photographed under inverted microscope (Nikon, Tokyo, Japan). The cells protruding from the border of the scratches were counted.

### Transwell invasion assay

Plates with transwell inserts (Corning, New York, USA) which were pre-coated with 20 μg/μL Matrigel (BD Biosciences, New Jersey, USA) were used to test cell invasion. The upper chambers with 200 μL serum-free media were added with 50,000 siRNA or lentivirus transfected cells. In parallel, 900 μL DMEM media with 10% FBS was added to lower chamber of each well. After incubation for 24 h at 37°C, cells from the upper surface of the membrane were removed with a cotton swab and the penetrated cells were fixed with 4% methanol for 5 min and then stained with 0.1% crystal violet for 30 min. Six fields of cells were captured and counted randomly under inverted microscope (Nikon, Tokyo, Japan) at ×20 magnification in each well. Upper chambers without Matrigel were used to test cell migration.

### Flow cytometry analysis

SiRNA or lentivirus transfected cells were harvested and washed with PBS. Next, 75% ethanol was used to fix cells at −20°C overnight. Then, DNA in cells was stained by Hank's balanced salt solution including 50 mg/mL propidium iodide and 50 mg/mL RNaseA for 1 h at room temperature. Cell cycle of these cells was analyzed by Gallios flow cytometer (Beckman Countler).

### Immunoprecipitation

SiRNA or lentivirus transfected cells were collected and lysed using lysis buffer supplemented with PMSF. Then, equal amounts of protein was subjected was subjected to anti-CBX7 antibody or anti-HDAC2 antibody following overnight incubation at 4°C. Then, protein-antibody immunoprecipitates were collected by protein A/G plus-agarose (Santa Cruz Biotechnology, Texas, USA).

### Chromatin immunoprecipitation (ChIP), reverse transcription PCR (RT-PCR) and quantitative real-time PCR (q-PCR)

ChIP assays were conducted using reagents commercially obtained from Upstate Biotechnology and performed according to the manufacturer's instructions. Briefly, glioma cells were then fixed with formaldehyde for 15 min. Then cells were lysed in SDS lysis buffer, and the chromatin DNA was extracted and sonicated into 200–1000 bp fragments. Purified DNA was used for PCR amplification. Enrichments at target sites were compared with IgG group.

*CCNE1* promoter primer sequences: F: 5′-ACGTGACCGTTGTGAGTCAA-3′; R:5′-CAGAGAGAAGAAGAGAAAGCTGAT-3′. *CCNE1* primer sequences: F: 5′-AAGGAGCGGGACACCATGA-3′; R: 5′-ACGGTCACGTTTGCCTTCC-3′. *GAPDH* primer sequences: F: 5′-TGTGGGCATCAATGGATTTGG3′; R: 5′-ACACCATGTATTCCGGGTCAAT-3′.

### Immunofluorescence

Cells were incubated with anti-CBX7 and anti-Cyclin E1 overnight and then with Alexa 488- or 568-labeled anti-mouse or anti-rabbit IgG antibody (Thermo Fisher Scientific, Massachusetts, USA) 2 h at room temperature. After treated with DAPI (Beyotime Biotechnology, Jiangsu, China) for 10 min, cells were examined with Zeiss axiophot photomicroscope (Carl Zeiss AG, Jena, Germany).

### *In vivo* experiments and IHC

Animal experiments were approved by the Animal Management Rule of the Chinese Ministry of Health (documentation 55, 2001) and were in accordance with the approved guidelines and the experimental protocol of Nanjing Medical University. Luciferase stably expressed U87 cells were transfected with Lenti-CBX7 or Lenti-Ctrl. Twelve mice purchased from Cancer Institute of the Chinese Academy of Medical Science were randomly divided into CBX7 upregulated group or control group. To establish intracranial glioma model, each nude mouse was implanted with 1 × 10^6^ U87^Luciferase/CBX7^ or U87^Luciferase/Ctrl^ cells (day 0). The intensity of bioluminescence was positively correlated with the number of U87 cells. Tumor growth was assessed weekly by live animal bioluminescence imaging system. After four times testing (day 7, day 14, day 21 and day 28), the survival of mice was observed until day 60. Then mice were exposed to CO_2_ to achieve euthanasia. Brains tissue sections were incubated with antibodies including CBX7 and Cyclin E1.

### Statistical analysis

*T*-test was employed to analyze differences in each two-group comparison and one-way ANOVA was performed to determine the difference among at least three groups. Kaplan–Meier analysis was used to assess the survival rate of mice. Heat maps were performed with Gene Cluster 3.0 and Gene Tree View software. Kaplan–Meier analysis was used to assess the survival rate of patients and mice. KEGG pathway and GO analysis were performed via DAVID (http://david.abcc.ncifcrf.gov/). *P* < 0.05 was considered statistically significant.

## SUPPLEMENTARY MATERIALS FIGURES AND TABLES



## Abbreviations

CBX7: chromobox homolog 7
PRC1: polycomb repressive complex 1
PcG: polycomb group proteins
HADC2: htone deacetylase 2
LGG: low grade glioma
HGG: high grade glioma
GBM: glioblastoma
