# Supplementary material for: CBX7 is a glioma prognostic marker and induces G1/S arrest via the silencing of CCNE1
Source: Oncotarget. 2017 Mar 1;8(16):26637–47. doi: 10.18632/oncotarget.15789 (PMC5432285; doi:10.18632/oncotarget.15789)
Supplement: Supplementary file 1 [file oncotarget-08-26637-s001.pdf]

CBX7 is a glioma prognostic marker and induces G_1/S arrest via the silencing of *CCNE1*

Supplementary Materials

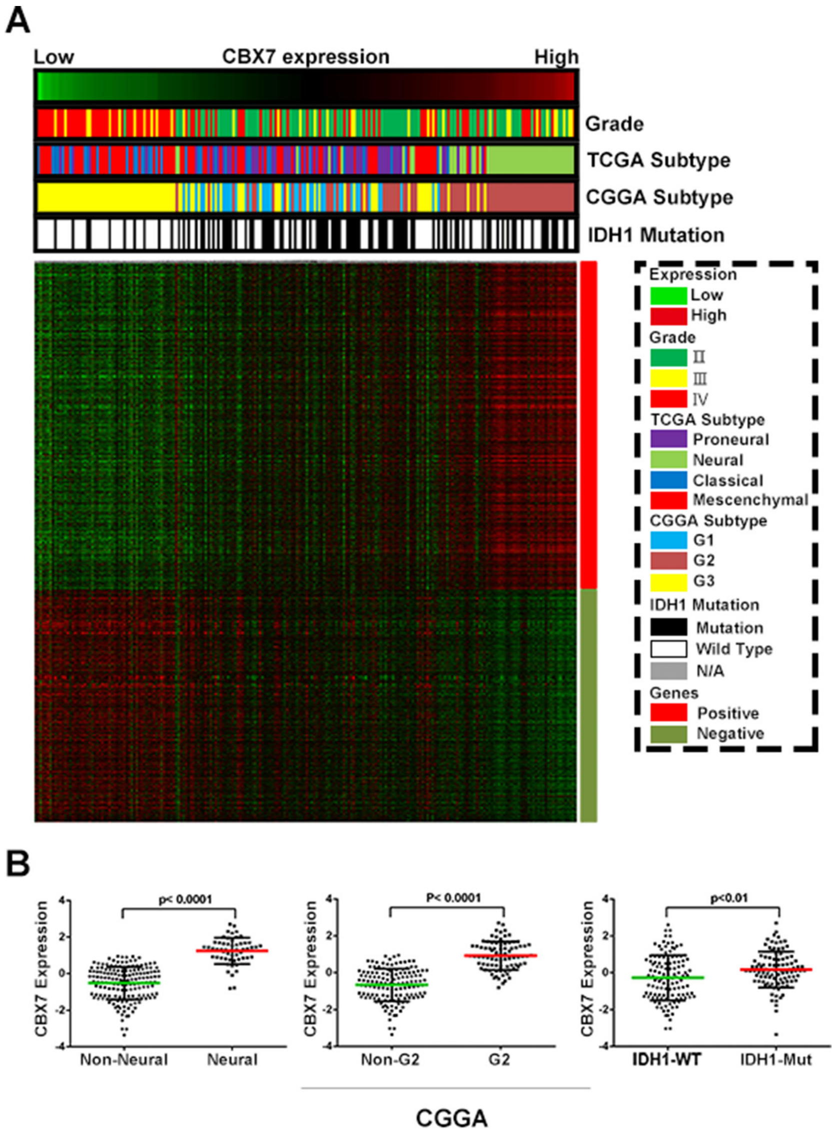

Supplementary Figure 1: CBX7 expression possesses subtype preferences in glioma. (**A**) Tumor grade, subtype of TCGA and CGGA, IDH1 mutation status were annotated and listed in the upper part. The lower part, a heat map of relative expression of CBX7-associated genes sorted by CBX7 expression level, illustrated the positively and negatively correlated genes. (**B**) Data from CGGA showed different neural, G2 subtypes and IDH1 mutation preferences.

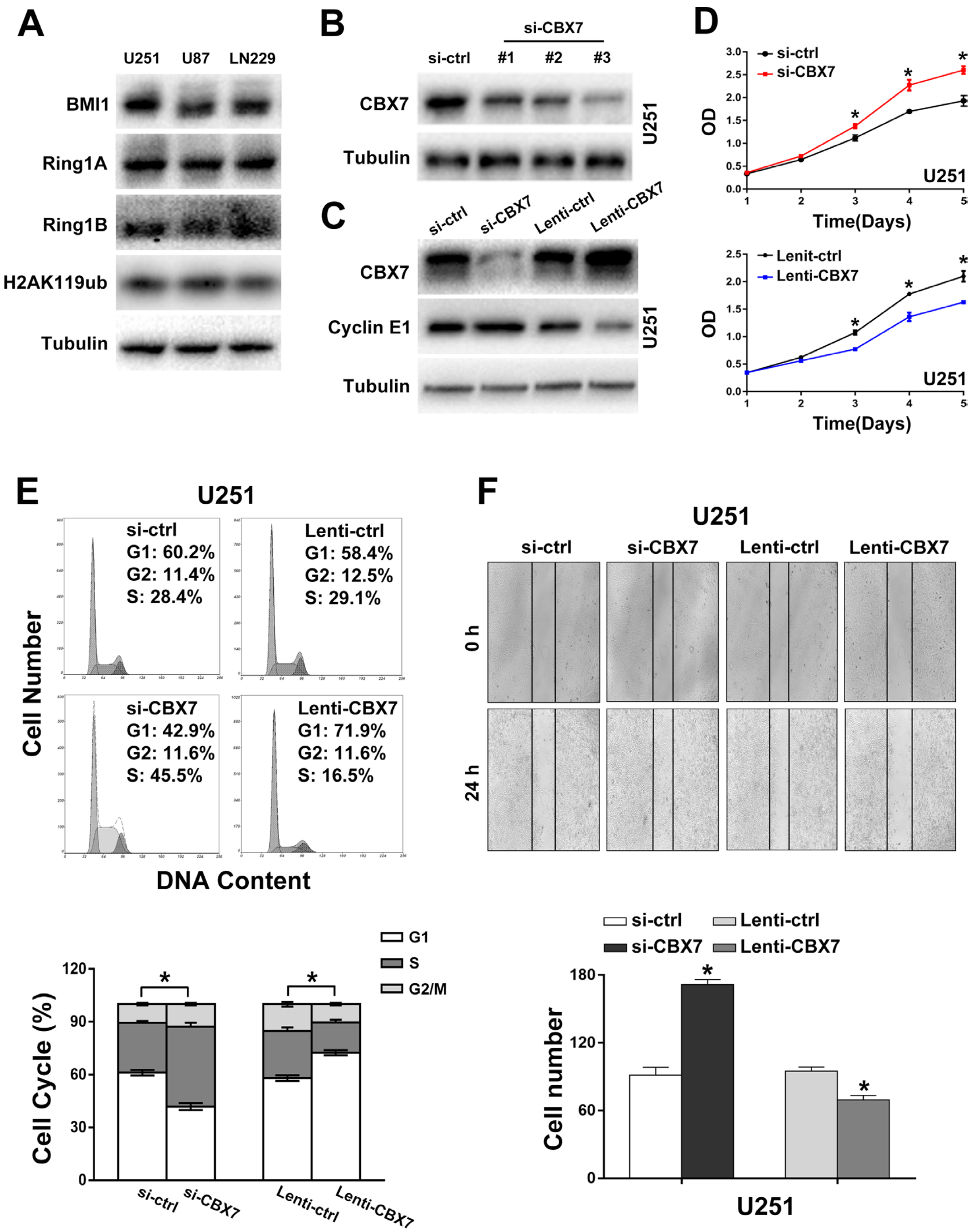

Supplementary Figure 2: The regulation of CBX7 influences proliferation, cell cycle and migration in U251 cells. (**A**) The protein levels of PRC1 complexes including BMI1, Ring1A, Ring1B and H2AK119ub in U251, U87 and LN229 cells were evaluated through western blot. (**B**) The efficiency of three different sequences si-CBX7 was tested in U251 cells and the optimal si-CBX7 sequences was chosen for the subsequent experiments. (**C**) After transient and stable transfection of si-CBX7 and lenti-CBX7 respectively in U251 cells, the expression of CBX7 and Cyclin E1 were tested by western blot. (**D**) The regulation of CBX7 via siRNA or lentivirus influenced the proliferation of U251 cells and the efficiency was determined by CCK8 assay. (**E**) Cell cycle progression of U251 cells which transfected with siRNAs or lentivirus was studied by flow cytometry. $P < 0.05$. (F) Migration ability in siRNAs- or lentivirus-transfected U251 cells was assessed by wound-healing assay and cells crossed the primary wound borders were counted. $P < 0.05$.

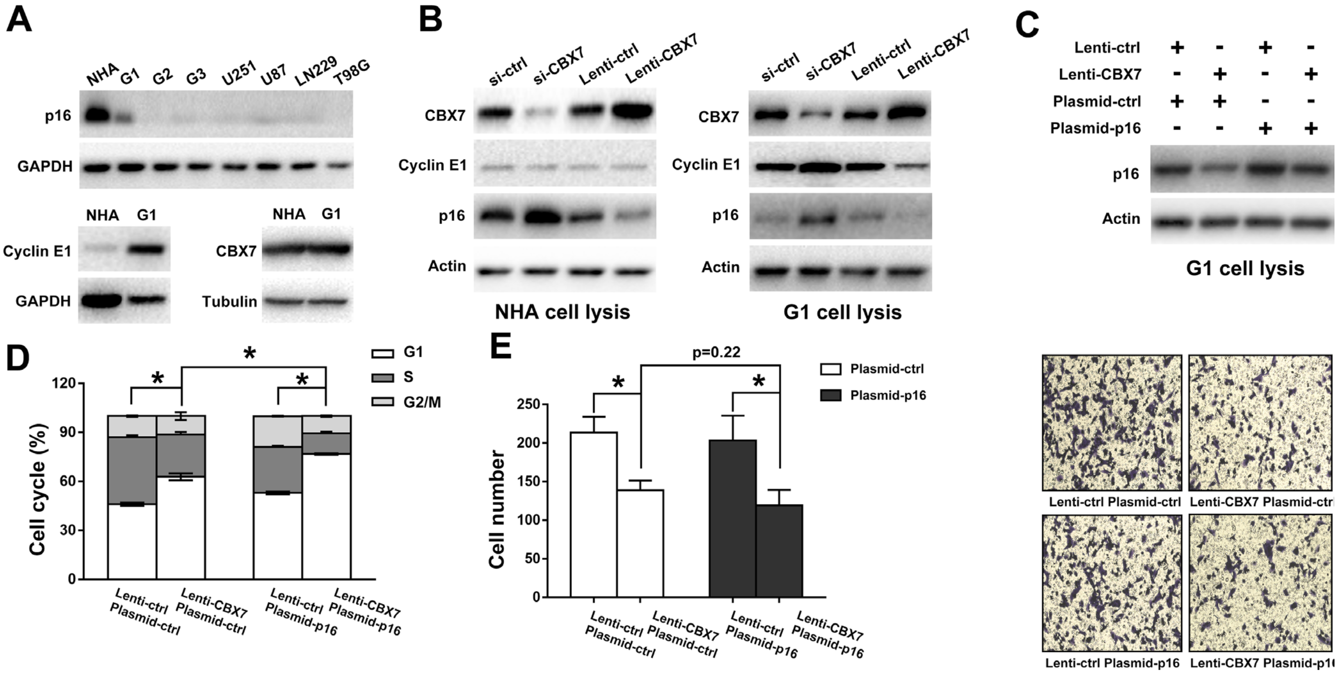

Supplementary Figure 3: The impairment of invasion in CBX7 overexpression glioma cells is p16-independent. (**A**) P16 protein levels in NHA, three kinds of primary GBM cells and four kinds of GBM cell lines were tested by western blot immunoassays. The expression of CBX7 and cyclin E1 in NHA and G1 cells were assessed and shown in down panel. (**B**) The alteration of CBX7, cyclin E1 and p16 expression in CBX7 regulated NHA and G1 cells were evaluated. (**C**) P16 levels in lenti-CBX7 G1 cells which were co-transfected of p16 expression plasmid (plasmid-p16) or empty plasmid (plasmid-ctrl) were evaluated by western blot. (D) Cell cycle status in co-transfected G1 cells was tested by flow cytometry. $P < 0.05$. (**E**) The right panel showed representative images of co-transfected G1 cells in transwell assay. Cells migrated through matrigel-coated surfaces were counted and illustrated in the left panel. $P < 0.05$.

Supplementary Table 1: General information of ten LGG patients

Characteristic	Value
**Histological Classification**	
astrocytoma tumor	8
oligodendroglioma	2
**Sex**	
Male	4
Female	6
**Medium age, years (range)**	43 (27–64)
**Tumor location**	
Frontal	5
Temporal	2
Others	3

General Information of ten HGG patients

Characteristic	Value
**Histological Classification**	
AA	6
GBM	4
**Sex**	
Male	5
Female	5
**Medium age, years (range)**	47 (24–72)
**Tumor location**	
Frontal	3
Temporal	4
Others	3
**MGMT promotor status**	
Methylated	7
Unmethylated	3

Abbreviations:
LGG, low grade glioma;
HGG, high grade glioma;
AA, anaplastic astrocytoma, grade III;
AO, anaplastic oligodendroglioma, grade III;
GBM, glioblastoma multiforme, grade IV;
MGMT, O-6-methylguanine-DNA-methyltransferase.
